# Supplementary material for: Habitat context affects sediment nitrogen burial by restored Eastern Oyster reefs
Source: PLoS One. 2026 Mar 25;21(3):e0344310. doi: 10.1371/journal.pone.0344310 (PMC13016323; doi:10.1371/journal.pone.0344310)

PLOS ONE

Habitat context affects sediment nitrogen burial by restored Eastern Oyster reefs

--Manuscript Draft--

**Manuscript Number:**	PONE-D-25-25624
**Article Type:**	Research Article
**Full Title:**	Habitat context affects sediment nitrogen burial by restored Eastern Oyster reefs
**Short Title:**	Nitrogen burial by restored oyster reefs
**Corresponding Author:**	Anne Smiley<br>The University of North Carolina at Chapel Hill<br>Chapel Hill, NC UNITED STATES OF AMERICA
**Keywords:**	Water quality; oysters; restoration; ecosystem services; nitrogen
**Abstract:**	Oysters perform essential functions in estuarine environments. Reef restoration has recently become the subject of significant attention to reestablish populations after historic losses and to restore valuable ecosystem functions and services, including nitrogen removal. Nitrogen burial in oyster reef sediments may be an important nitrogen sink, but direct measurements are lacking. We assayed sediments from 11- to 14-year-old restored oyster reefs in three representative habitat contexts in a temperate estuary on the US Atlantic Coast. Nitrogen burial rates ranged between 1.02 and 14.7 g N m-2 y-1 and generally scaled positively with reef relief and density. Intertidal flat reefs exhibited the greatest relief values, densities, and nitrogen burial rates. Subtidal flat reefs produced the lowest relief values and burial rates. Intertidal fringing reefs exhibited the lowest C:N ratios—burying proportionally more nitrogen than reefs in other habitat contexts. Using avoided cost methods, the value of nitrogen burial by oyster reefs in all habitat contexts ranged from $270 to $3,900 US dollars (USD) per hectare per year with an average of $1,700 USD per hectare per year. Integrating this figure into current estimates of nitrogen removal ecosystem services would increase the value 25-42%. Our findings suggest that specific site selection for restored and protected reefs can maximize nitrogen removal through multiple mechanisms, including burial. Providing empirical measurements of ecosystem function and estimates of economic value can inform site selection and design of restored oyster reefs to maintain water quality.
**Order of Authors:**	Anne Margaret Smiley, PhD
	Joel Fodrie, PhD
	Jonathan H. Grabowski, PhD
	Antonio B. Rodriguez, PhD
	Suzanne P. Thompson
	Michael F. Piehler, PhD
**Additional Information:**	
**Question**	**Response**
**Financial Disclosure**<br><br>Enter a financial disclosure statement that describes the sources of funding for the work included in this submission. Review the submission guidelines for detailed requirements. View published research articles from *PLOS ONE* for specific examples.	Yes

Powered by Editorial Manager® and ProduXion Manager® from Aries Systems Corporation

This statement is required for submission and **will appear in the published article** if the submission is accepted. Please make sure it is accurate.<br><br>**Funded studies**<br>Enter a statement with the following details:<br>• Initials of the authors who received each award<br>• Grant numbers awarded to each author<br>• The full name of each funder<br>• URL of each funder website<br>• Did the sponsors or funders play any role in the study design, data collection and analysis, decision to publish, or preparation of the manuscript?<br><br>Did you receive funding for this work?	
Please add funding details.<br>as follow-up to "**Financial Disclosure**<br><br>Enter a financial disclosure statement that describes the sources of funding for the work included in this submission. Review the submission guidelines for detailed requirements. View published research articles from *PLOS ONE* for specific examples.<br><br>This statement is required for submission and **will appear in the published article** if the submission is accepted. Please make sure it is accurate.<br><br>**Funded studies**<br>Enter a statement with the following details:<br>• Initials of the authors who received each award<br>• Grant numbers awarded to each author<br>• The full name of each funder<br>• URL of each funder website<br>• Did the sponsors or funders play any role in the study design, data collection and analysis, decision to publish, or preparation of the manuscript?<br><br>Did you receive funding for this work?"	This research was supported by the UNC Institute for the Environment, the National Science Foundation (OCE-1233327, OCE-1155628, OCE-1635950), the United States Coastal Research Program (W912HZ-22-COO-11), and North Carolina Sea Grant (12-HCE-20).

Powered by Editorial Manager® and ProduXion Manager® from Aries Systems Corporation

Please select the country of your main research funder (please select carefully as in some cases this is used in fee calculation).<br>as follow-up to "**Financial Disclosure**<br><br>Enter a financial disclosure statement that describes the sources of funding for the work included in this submission. Review the submission guidelines for detailed requirements. View published research articles from *PLOS ONE* for specific examples.<br><br>This statement is required for submission and **will appear in the published article** if the submission is accepted. Please make sure it is accurate.<br><br>**Funded studies**<br>Enter a statement with the following details:<br>• Initials of the authors who received each award<br>• Grant numbers awarded to each author<br>• The full name of each funder<br>• URL of each funder website<br>• Did the sponsors or funders play any role in the study design, data collection and analysis, decision to publish, or preparation of the manuscript?<br><br>Did you receive funding for this work?"	UNITED STATES - US
**Competing Interests**<br><br>Use the instructions below to enter a competing interest statement for this submission. On behalf of all authors, disclose any competing interests that could be perceived to bias this work—acknowledging all financial support and any other relevant financial or non-financial competing interests.<br><br>This statement is required for submission and **will appear in the published article** if the submission is accepted. Please make sure it is accurate and that any funding	The authors have declared that no competing interests exist.

sources listed in your Funding Information later in the submission form are also declared in your Financial Disclosure statement.<br><br>View published research articles from *PLOS ONE* for specific examples.<br><br>**NO authors have competing interests**<br><br>Enter: *The authors have declared that no competing interests exist.*<br><br>**Authors with competing interests**<br><br>Enter competing interest details beginning with this statement:<br><br>*I have read the journal's policy and the authors of this manuscript have the following competing interests: [insert competing interests here]*<br><br>* typeset	
**Ethics Statement**<br><br>Enter an ethics statement for this submission. This statement is required if the study involved:<br><br>• Human participants<br>• Human specimens or tissue<br>• Vertebrate animals or cephalopods<br>• Vertebrate embryos or tissues<br>• Field research<br><br>Write "N/A" if the submission does not require an ethics statement.<br><br>General guidance is provided below. Consult the submission guidelines for detailed instructions. **Make sure that all information entered here is included in the Methods section of the manuscript.**	N/A

**Format for specific study types**<br><br>**Human Subject Research (involving human participants and/or tissue)**<br>• Give the name of the institutional review board or ethics committee that approved the study<br>• Include the approval number and/or a statement indicating approval of this research<br>• Indicate the form of consent obtained (written/oral) or the reason that consent was not obtained (e.g. the data were analyzed anonymously)<br><br>**Animal Research (involving vertebrate animals, embryos or tissues)**<br>• Provide the name of the Institutional Animal Care and Use Committee (IACUC) or other relevant ethics board that reviewed the study protocol, and indicate whether they approved this research or granted a formal waiver of ethical approval<br>• Include an approval number if one was obtained<br>• If the study involved *non-human primates*, add *additional details* about animal welfare and steps taken to ameliorate suffering<br>• If anesthesia, euthanasia, or any kind of animal sacrifice is part of the study, include briefly which substances and/or methods were applied<br><br>**Field Research**<br><br>Include the following details if this study involves the collection of plant, animal, or other materials from a natural setting:<br>• Field permit number<br>• Name of the institution or relevant body that granted permission	
**Data Availability**<br><br>Authors are required to make all data underlying the findings described fully available, without restriction, and from the time of publication. PLOS allows rare exceptions to address legal and ethical concerns. See the PLOS Data Policy and FAQ for detailed information.	Yes - all data are fully available without restriction

A Data Availability Statement describing where the data can be found is required at submission. Your answers to this question constitute the Data Availability Statement and **will be published in the article**, if accepted.<br><br>**Important:** Stating 'data available on request from the author' is not sufficient. If your data are only available upon request, select 'No' for the first question and explain your exceptional situation in the text box.<br><br>Do the authors confirm that all data underlying the findings described in their manuscript are fully available without restriction?	
**Describe where the data may be found in full sentences. If you are copying our sample text, replace any instances of XXX with the appropriate details.**<br><br>• If the data are **held or will be held in a public repository**, include URLs, accession numbers or DOIs. If this information will only be available after acceptance, indicate this by ticking the box below. For example: *All XXX files are available from the XXX database (accession number(s) XXX, XXX.).*<br>• If the data are all contained **within the manuscript and/or Supporting Information files**, enter the following: *All relevant data are within the manuscript and its Supporting Information files.*<br>• If neither of these applies but you are able to provide **details of access elsewhere**, with or without limitations, please do so. For example:<br><br>*Data cannot be shared publicly because of [XXX]. Data are available from the XXX Institutional Data Access / Ethics Committee (contact via XXX) for researchers who meet the criteria for access to confidential data.*<br><br>*The data underlying the results presented in the study are available from (include the name of the third party*	All data files are available in the Carolina Digital Repository (https://cdr.lib.unc.edu/concern/data_sets/gq67k604s).

and contact information or URL).<br>• This text is appropriate if the data are owned by a third party and authors do not have permission to share the data.<br><br>* typeset	
Additional data availability information:	Tick here if the URLs/accession numbers/DOIs will be available only after acceptance of the manuscript for publication so that we can ensure their inclusion before publication.

Powered by Editorial Manager® and ProduXion Manager® from Aries Systems Corporation

Manuscript

Click here to access/download;Manuscript;Habitat context nitrogen burial restored oyster reefs.docx

Habitat context affects sediment nitrogen burial by restored Eastern Oyster reefs

Anne Margaret H. Smiley[1,2], F. Joel Fodrie[2], Jonathan H. Grabowski[3], Antonio B. Rodriguez[2], Suzanne P. Thompson[1,2], and Michael F. Piehler[1,2]

[1] Institute for the Environment, University of North Carolina, Chapel Hill, NC 27514, USA

[2] Department of Earth, Marine, and Environmental Sciences, University of North Carolina, 5 Chapel Hill, NC 27514, USA

[3] Department of Marine and Environmental Sciences, Northeastern University, Boston, MA 02115, USA

Abstract

Oysters perform essential functions in estuarine environments. Reef restoration has recently become the subject of significant attention to reestablish populations after historic losses and to restore valuable ecosystem functions and services, including nitrogen removal. Nitrogen burial in oyster reef sediments may be an important nitrogen sink, but direct measurements are lacking. We assayed sediments from 11- to 14-year-old restored oyster reefs in three representative habitat contexts in a temperate estuary on the US Atlantic Coast. Nitrogen burial rates ranged between 1.02 and 14.7 g N m^{-2} y^{-1} and generally scaled positively with reef relief and density. Intertidal flat reefs exhibited the greatest relief values, densities, and nitrogen burial rates. Subtidal flat reefs produced the lowest relief values and burial rates. Intertidal fringing reefs exhibited the lowest C:N ratios—burying proportionally more nitrogen than reefs in other habitat contexts. Using avoided cost methods, the value of nitrogen burial by oyster reefs in all habitat contexts ranged from $270 to $3,900 US dollars (USD) per hectare per year with an average of $1,700 USD per hectare per year. Integrating this figure into current estimates of nitrogen removal ecosystem services would increase the value 25-42%. Our findings suggest that specific site selection for restored and protected reefs can maximize nitrogen removal through multiple mechanisms, including burial. Providing empirical measurements of ecosystem function and estimates of economic value can inform site selection and design of restored oyster reefs to maintain water quality.

1. Introduction

Oysters provide essential contributions to ecosystem function through the provision of habitat and nursery areas, sediment stabilization, and nutrient cycling. These functions provide ecosystem services that directly benefit coastal communities by supporting economically important fisheries (Scyphers et al., 2011), reducing damage during storm events (Hynes et al., 2022; Scyphers et al., 2011), and maintaining water quality (Barbier et al., 2011; Coen et al., 2007; Mehvar et al., 2018; Piehler & Smyth, 2011). Increased oyster reef restoration in recent decades has attempted to reestablish populations after historic losses—up to 85% globally (Beck et al., 2011)—and restore valuable ecosystem functions and services (Bersoza Hernández et al., 2018). Furthermore, oysters' ability to capture, produce, and retain sediments to maintain their vertical position in the water column, make them uniquely resilient to sea level rise (Ridge et al., 2017; Rodriguez et al., 2014).

Passive accumulation of allogenic materials coupled with active organic matter generation through deposition of feces and pseudofeces leads to enhanced levels of sediment nutrient concentrations (Chambers et al., 2017; Newell et al., 2005) and can be an important regulator of water quality. Physical and biogeochemical processes in the shallow sediments, or the taphonomically active zone (TAZ), and subsequent burial can influence availability of nitrogen (N) for bloom-forming phytoplankton. Microbial decomposition of organic nitrogen

produces inorganic, bioavailable forms of nitrogen that may be removed via bioassimilation (Carmichael et al., 2012; Higgins et al., 2011) or denitrification (Piehler & Smyth, 2011; Rosenzweig et al., 2018; Seitzinger, 1987; Smyth et al., 2015; Velinsky et al., 2017), or otherwise efflux out of the sediments (Burdige & Zheng, 2003). Nitrogen compounds that evade these processes are buried below the TAZ, where they are no longer bioavailable.

Burial in oyster reef sediments could be an important sink for nitrogen (Beseres Pollack et al., 2013; Newell et al., 2005), but direct measurements of burial rates in these habitats are lacking. Nitrogen burial rates have been measured in tidal marshes and estuarine bay sediments in multiple ecoregions, including the US Atlantic Coast (Boynton & Kemp, 1985; Craft, 2007; Loomis & Craft, 2010; Palinkas & Cornwell, 2011) and Gulf Coast (DeLaune et al., 1981; Smith et al., 1985; Westbrook et al., 2018), generally ranging between 0-25 g N $m^{-2}y^{-1}$. Westbrook et al. (2018) measured nitrogen burial in shallow- and deep-water oyster reefs in a Gulf Coast estuary and reported rates of 1.4-2.6 g N m^{-2} y^{-1}. However, regional differences in environmental conditions—including wave action, tidal range, and harvest practices—alter oyster reef morphology (Byers et al., 2015) and growth, and likely nitrogen burial.

Local environmental conditions can also influence oyster reef properties and functions (Keller et al., 2019). Reef relief and vertical position in the water column can affect oyster recruitment (O'Beirn et al., 2000; Perog et al., 2023; Schulte et al., 2009), and potentially reef assimilation of nitrogen and/or production of nitrogen-rich biodeposits. Exposure to high flow velocities has a strong influence on reef size and complexity (Bahr & Lanier, 1981), and surface area for sediment capture. Proximity to other estuarine habitats, such as marshes, can influence sediment flux and composition (Ridge et al., 2017), and consequently biogeochemical processing in the TAZ. Physicochemical characteristics of the water column also influences biogeochemical cycling; elevated nitrate concentrations can increase direct denitrification rates in estuarine habitats, including oyster reef sediments (Smyth et al., 2015), affecting the nitrogen pool available to be buried below the TAZ. In urban estuaries, where anthropogenic nitrogen loads are often elevated, oyster reef restoration may be an effective strategy to regain and/or maintain water quality (Coen et al., 2007; Kellogg et al., 2014; Onorevole et al., 2018; Rose et al., 2014; Smyth et al., 2012).

Oyster restoration can be costly (Bayraktarov et al., 2016; Hall & DeAngelis, 2024), and comprehensively accounting for ecosystem services provided by oyster reefs can help to quantify returns on investment and strategically design restoration projects (BenDor et al., 2017; Grabowski et al., 2012). Oysters have marketplace value through productive fisheries, but this alone does not capture the non-market value provided through additional ecosystem functions, such as nitrogen removal. Grabowski et al. (2012) estimated that oyster reefs provide between $5,000 and $100,000 worth of ecosystem services per hectare annually, with nearly 40% of the total non-market value attributed to nitrogen removal on average. However, these figures do not include nitrogen removal through burial.

The objectives of this study were (1) to quantify sediment nitrogen content and burial rates in restored oyster reefs in varied habitat contexts in a temperate estuary on the US Atlantic Coast, and (2) determine whether restored oyster reefs bury nitrogen at economically and environmentally relevant rates. To do this, we assayed sediments from 11- to 14-year-old restored oyster reefs in 3 representative estuarine habitat contexts: intertidal flat, subtidal flat, and intertidal fringing reef. We also calculated the value of nitrogen removal from burial using an established nutrient credit trading system, and compared nitrogen burial rates to nitrogen loads from a municipal wastewater treatment facility. Though the capacity of restored oyster reefs to bury N has not been measured before in US Atlantic Coast estuaries, it is critical for a complete understanding of the ecosystem functioning and economic values of restored oyster reefs in particular and oyster reefs in general.

2. Methods

2.1 Study sites

In 2011, we sampled 20 experimental oyster reefs that were created in 1997 or 2000 in Back Sound, NC, USA (Figure 1) using approximately 60 bushels of oyster shell to achieve dimensions of roughly 5 x 3 x 0.30 m (Grabowski et al., 2022; Grabowski et al., 2005), representative of natural reef sizes in this region (Bahr & Lanier, 1981). The reefs are scattered around Middle Marsh, a setting characterized by salt marshes, seagrass beds, and sand/mud flats, experiencing semi-diurnal tides that range approximately 0.9 m (Ridge et al., 2017; Rodriguez et al., 2014). They are located within the Rachel Carson National Estuarine Research Reserve and have been protected from harvest since their construction. We sampled reefs across habitat contexts and inundation regimes, including intertidal flat (n = 7), subtidal flat (n = 3), and intertidal adjacent to saltmarsh hereafter referred to as intertidal fringing reef (n = 10). Reefs considered in a "flat" habitat context are within a mudflat or sandflat. The two westernmost subtidal flat reefs are seasonally surrounded by seagrass.

Fig 1. Site map of 20 sampled oyster reefs in Middle Marsh, Back Sound, NC, USA.

2.2 Reef properties

2.2.1 Relief

We surveyed the reefs and surrounding areas using a Trimble Real Time Kinematic Global Positioning System mounted on a 2.0 m staff with an average vertical precision of 1.5 cm. Survey points were collected about every 50 cm along two reef-crossing transects. Transects across the patch reefs were oriented perpendicular to each other and crossed at the reef center. The transects across the fringing reefs were oriented parallel and perpendicular to the trend of the marsh shoreline and crossed near the edge of the salt marsh. The reefs are flat on top with steep sides; therefore, most of the survey points were obtained either from areas on top of the reef or on the adjacent sand flat. We defined reef relief as the difference between the average elevation of the reef top, based on 5 to 12 observations (± 1 SD), and the average elevation of the adjacent sandflat based on 4 to 10 observations (± 1 SD).

2.2.2 Density

To estimate oyster densities across each reef in which nitrogen burial estimates were made, we located two random 0.25-m^2 quadrats on intertidal flat and intertidal fringing reefs. Density data were unavailable for subtidal flat reefs. The top 10 cm of material within each 0.25-m^2 quadrat was excavated. Samples were sieved though a 1-mm mesh, and every live oyster was counted and measured to the nearest mm from the hinge to the leading growth margin.

2.2.3 Sediment N and C contents

To quantify the nitrogen composition of reefs, we drove 10 cm diameter aluminum pipes vertically through the X-Y center of each oyster reef using a gas-powered jack hammer. Cores sampled the entire reef structure (10-55 cm deep) and a few decimeters of the underlying substrate. Sediments were then ground, fumed with 1NM HCl, and re-dried prior to induction in a Perkin Elmer CHN analyzer (Model 2400) to determine % nitrogen and % carbon (carbon data reported in Fodrie et al. 2017). Bulk-weight and % nitrogen data of sediments were combined to quantify nitrogen (by weight, g) in each core section (5 cm). Measurements of nitrogen were vertically integrated to produce estimates of total nitrogen, or lifetime burial, in reefs (g N m^{-2}). To calculate burial rates, we divided the overall weight of nitrogen within each down-core sample by the age of each experimental oyster reef.

2.3 Statistical Methods

Several complimentary analyses were performed to explore patterns and correlates of nitrogen burial within restored experimental oyster reefs located on intertidal sand flats, subtidal sand flats, or adjacent to salt marshes within Middle Marsh.

First, we used linear regression analysis with data from all 20 experimental reefs to consider the relationship between total nitrogen content (lifetime burial) within oyster reefs (g N m^{-2}) and the vertical relief of each reef (i.e., upward accretion; in cm). Next, a polynomial regression was used to examine the link between live oyster density (individuals m^{-2}) and nitrogen burial rate (g N m^{-2} yr^{-1}) at intertidal sand flat and fringing reefs. A second order fit was chosen following visual inspection of the X-Y scatterplot. Since density data were unavailable for subtidal sand flat reefs, they were excluded from this analysis. Reef-scale densities were based on the average of the two quadrats sampled from each reef. In these analyses, we considered nitrogen content and burial rates the dependent variables.

We utilized one-way ANOVAs to test for effects of habitat context on nitrogen content (g N m^{-2}) and reef densities (# m^{-2}) within the 11-to-14-year-old experimental oyster reefs. These data were tested for normality using Shapiro-Wilk tests across the main effect of habitat context, and no transformations were required to satisfy the assumptions of parametric analyses. Based on a significant main effect ($\alpha < 0.05$), we used Tukey HSD tests for post-hoc comparisons between treatments. Nitrogen burial rates (g N m^{-2} y^{-1}), C:N ratios, and relief (cm) data violated the assumption of normality; therefore, Kruskal Wallis and post hoc Dunn tests were used to compare means. Finally, the linear relationship between % carbon and % nitrogen within

sediments was examined using regression analysis. For these analyses, we used the CHN outputs from each 5-cm core section as individual data points.

2.4 Economic valuation and environmental relevance of N removal service

We extrapolated our measurements across reef area within Carteret County, NC according to NC Department of Marine Fisheries' Estuarine Benthic Habitat Mapping Program, Iteration 2, to evaluate prevalence of reefs in each habitat context and to estimate the amount and value of nitrogen burial at a local scale. Coastal counties in NC comply with the State's Coastal Area Management Act (15A North Carolina Administrative Code 07B), playing an important role in estuarine habitat management. We further rationalize a county-wide assessment due to the availability of data at this scale (zu Ermgassen et al., 2021).

To estimate annual nitrogen removal rates by oyster reefs (kg N y^{-1}), burial rates for each habitat context were multiplied by the total reef area, which was determined considering only shell-bottom features. Features classified as "intertidal firm non-vegetated", or "intertidal hard non-vegetated" bottom were used to delineate intertidal sand flat reefs. Features labeled as "subtidal firm non-vegetated", or "subtidal hard non-vegetated" bottom were combined to identify subtidal sandflat reefs. Intertidal fringing reefs were delineated using features classified as "intertidal firm vegetated" or "intertidal hard vegetated" bottom. Substrates that were classified as "soft" were excluded from this spatial analysis. The habitat survey protocol does not distinguish between natural and restored reefs; however, Chambers et al. (2017) found that restored intertidal reefs rapidly increase sediment nutrient concentrations and are comparable with older restored reefs and natural reefs within a year of construction. Therefore, we assumed that measurements collected from the restored reefs in Back Sound are representative of natural reefs that are closed to harvest, including those located downstream of a municipal wastewater treatment facility.

We repeat the process for a subset of the Carteret County's estuarine waters in Calico Creek. Oyster reefs in Calico Creek are located downstream of Morehead City's wastewater treatment facility (NPDES permit #NC002661 or #NCG110110) and are closed to harvest due to chronically high concentrations of pollutants. Annual nitrogen burial rates were compared to dissolved inorganic nitrogen (DIN) inputs from this anthropogenic nitrogen point source. Annual DIN loads were obtained from the Environmental Protection Agency's Enforcement and Compliance History Online (ECHO) database (https://echo.epa.gov). The average annual loading rate was calculated by taking the average of values reported between 2013-2015 and 2020-2023—no values were reported from 2016-2019 in the ECHO database; therefore, those years were excluded from this analysis.

The economic value of nitrogen removal through burial by oyster reefs was calculated using rate schedules established in North Carolina's Nutrient Offset Program (15A North Carolina Administrative Code 2B.0703; Piehler & Smyth, 2011). The NC Department of Environmental Quality (DEQ) valued one kilogram of nitrogen removed at $26.39 in 2024 US

dollars (USD) in the Tar-Pamlico basin. This figure was multiplied by annual nitrogen removal rates for each habitat context, which integrates the fine-scale nitrogen burial measurements and the spatial coverage of that type of reef.

3. Results

3.1 Sediment N across habitat contexts

Total nitrogen composition quantified for each reef revealed that lifetime nitrogen burial ranged from 13.3 to 162 g N m^{-2} with a mean of 78.8 ± 9.6 g N m^{-2} across habitat contexts. Total nitrogen content exhibited a significant positive linear relationship with reef relief ($R^2 = 0.67$, $p < 0.001$; Figure 2A). Nitrogen burial rates were calculated for each of the 20 restored oyster reefs and ranged between 1.03 and 14.7 g N m^{-2} y^{-1} with a mean of 6.42 ± 0.82 g N m^{-2} y^{-1}. A significant parabolic relationship was identified between live oyster density and nitrogen burial rates in intertidal sand flat and fringing reefs ($p < 0.001$; Figure 2B). The second order polynomial fit predicted maximum burial rates to occur at reef densities of 3,000 live oysters m^{-2}.

Fig 2. Relationships between oyster-reef vertical relief (cm) and total nitrogen stored within oyster reefs (g N m^{-2}; A); and live oyster density (individuals m^{-2}) and annually averaged nitrogen burial rates by intertidal restored oyster reefs (g N m^{-2} y^{-1}; B).

Habitat context had a significant effect on reef relief, live oyster density, nitrogen composition, and nitrogen burial rates (Figure 3). Intertidal sand flat reefs had significantly greater mean relief (50.9 ± 4.1 cm) than either to subtidal flat or intertidal fringing reefs (16.7 ± 3.4 cm and 22.1 ± 3.9 cm, respectively). Live oyster densities in intertidal flat reefs were 874 ± 118 m^{-2}, which was significantly greater than those in intertidal fringing reefs (111 ± 16 m^{-2}). Intertidal flat reefs also contained the greatest amount of nitrogen with 110 ± 9 g N m^{-2}, which was significantly higher than lifetime burial in subtidal sand flat reefs (38.0 ± 11.6 g N m^{-2}). Lifetime burial in intertidal fringing reefs (69.4 ± 14.2 g N m^{-2}) was slightly lower than in intertidal sand flat reefs, but this difference was not statistically significant ($p = 0.08$). Nitrogen burial rates were significantly higher in intertidal sand flat reefs (9.03 ± 0.74 g N m^{-2} y^{-1}) than in subtidal sand flat and intertidal fringing reefs (3.57 ± 1.06 g N m^{-2} y^{-1} and 5.45 ± 1.26 g N m^{-2} y^{-1}, respectively; Figure 3).

Fig 3. Mean values across habitat contexts for relief (cm; A), density (individuals m-2; B), N composition (g N m-2; C), and burial rate (g N m-2 y-1; D).

For all restored oyster reefs, % carbon scaled positively with % nitrogen ($R^2 = 0.742$; Figure 4), with intertidal sand flat reef sediments containing the highest amounts carbon and nitrogen compared to other reefs. Significant linear relationships between % carbon and % nitrogen in reef sediments were evident for each individual habitat context. Average C:N ratios significantly differed across habitat contexts, with intertidal fringing reef sediments producing significantly lower values than those in either intertidal or subtidal sand flat reefs (Table 1).

Fig 3. Linear regression to compare % nitrogen and % carbon in oyster reef sediments for each habitat context. Each datum represents a 5-cm vertical section of a core collected from oyster reefs. Analysis produced an R^2 value of 0.742.

Table 1. Summary of mean C:N ratio across habitat contexts, calculated using values from each core section. Superscripts indicate statistical significance.

Habitat context	n	C:N ratio
Intertidal flat	68	22.0 ± 0.9[a]
Subtidal flat	17	27.0 ± 3.8[a]
Intertidal fringing	54	18.5 ± 1.3[b]

3.2 Economic valuation of nitrogen burial

Using the mean nitrogen burial rate across all habitat contexts, we estimated that the average annual value of nitrogen burial by oyster reefs is $1,720 USD per hectare. In Carteret County, there are approximately 274 hectares of intertidal sand flat oyster reefs, 2400 hectares of subtidal sand flat oyster reefs, and 166 hectares of intertidal fringing oyster reefs. Annually, Carteret County oyster reefs removed roughly 120,000 kg of nitrogen through burial valued at approximately $3,160,000 USD. Subtidal sand flat oysters comprise the largest portion at around $2,260,000 USD, followed by intertidal sand flat reefs at ~$653,000 USD and intertidal fringing reefs at ~$244,000 USD.

We also report nitrogen burial and economic value of this ecosystem service provided by oysters in Calico Creek in Morehead City, NC (Figure 5). This tidal creek hosts oyster reefs of all three habitat contexts investigated in this study and is affected by wastewater effluent, affording the opportunity to relate removal through burial to input from a nitrogen point source. In Calico Creek, there are roughly 28.9 hectares of oyster reefs comprised of 19.2 hectares of intertidal sand flat, 3.5 hectares of subtidal sand flat, and 6.2 hectares of intertidal fringing reefs. Collectively, these reefs remove approximately 2,200 kg N yr^{-1} through burial. The Morehead City wastewater treatment facility adds roughly 10,900 kg DIN yr^{-1} to Calico Creek; therefore, oysters in this system potentially remove 20% of the wastewater treatment facility's DIN load.

Fig 4. Map of oyster reefs in Calico Creek, NC, USA, as delineated by the NC Department of Marine Fisheries. Average nitrogen burial rates for each habitat context were multiplied by the area of respective reefs to estimate annual removal and value of the ecosystem service.

4. Discussion

This is the first study to directly measure nitrogen burial rates in oyster reefs in a temperate estuary on the US Atlantic Coast. Compared to other structured estuarine habitats in this ecoregion, like marshes and seagrasses, oyster reefs can bury nitrogen at considerably higher

rates. Marshes in coastal Georgia, USA accumulated nitrogen at rates up to 6.9 g N m^{-2} y^{-1} (Craft, 2007; Loomis & Craft, 2010). Aoki et al. (2019) reported nitrogen burial rates in restored seagrass meadows in South Bay, VA up to 3.5 g-N m^{-2}. The complexity/rugosity of oyster reefs compared to vegetated habitats may promote sediment capture and explain why the upper threshold measured in the restored oyster reefs was more than double the values recorded in the marsh and more than four times those in seagrasses.

Nitrogen burial by oysters was comparable to denitrification rates measured in oyster reefs in the same system. Smyth et al. (2012) reported average annual denitrification rates in Bogue Sound oyster reefs between 5-7 g N m^{-2} y^{-1}. Bioassimilation of nitrogen into oyster tissues and subsequent harvest is another potential nitrogen removal mechanism in oyster aquaculture operations. Beseres Pollack et al. (2013) estimated that approximately 18 km^2 of oyster reefs in the Mission-Aransas Estuary can remove over 21,000 kg N y^{-1}, or roughly 1.2 g N m^{-2} y^{-1}, through harvest. Nitrogen removal via burial by Back Sound oysters is over 5 times higher than this value.

4.1 Regional scale environmental context affects nitrogen burial

Nitrogen burial rates measured in this study were higher than those measured in coastal Louisiana (LA) reefs by Westbrook et al. (2018), which ranged from 1.4-2.6 g N m^{-2} y^{-1}. Regional differences in environmental conditions and external harvest pressure affect oyster reef morphology (Byers et al., 2015) and may explain this difference. East Coast oyster reefs are generally distributed in denser more well-defined patches, whereas Gulf Coast reefs are more scattered and sparser. Westbrook et al. (2018) and Casas et al. (2015) report maximum densities of 500 individuals m^{-2}—an order of magnitude lower than the maximum density observed in Back Sound. Back Sound, NC experiences semidiurnal tides ranging roughly 1 m, whereas Sister Lake, one of the sites included in the Westbrook study, experiences diurnal tides ranging only 0.3 m. Higher flow velocities and energy can mix the water column and increase the food supply to oysters to enhance their growth (Housego & Rosman, 2015; Lenihan et al., 1996) and production of biodeposits. Exposure to high flow velocities can also increase reef complexity (Byers et al., 2015), which may enhance the capture and retention of allogenic materials to supplement the biogenic sediment supply and promote vertical reef building.

Differences in harvest pressure may also explain differences in nitrogen burial rates in LA and NC oyster reefs. Nitrogen burial is ultimately reliant on a reef's ability to vertically accrete, and reef relief and density generally scaled positively with burial rates. Thus, active removal of individuals may hamper the capacity to bury nitrogen. The LA reefs examined in the Westbrook et al. (2018) study are subject to active harvest, potentially explaining the low nitrogen burial rates compared to the protected reefs in Middle Marsh, NC. This information has implications for reefs that are closed to harvest for conservation, fishery repopulation, and/or chronic exposure to pollutants.

4.2 Local scale environmental context affects nitrogen burial

Habitat context at the local scale had a significant effect on oyster reef characteristics and nitrogen burial rates. We found that intertidal sand flat reefs had greater relief and buried nitrogen at significantly higher rates than subtidal sand flat reefs. Position within the tidal frame can impact growth of oysters as some percentage of aerial exposure can reduce biofouling and predation from pelagic species (Fodrie et al., 2014). Ridge et al. (2015) identified the optimal growth zone (OGZ) for oysters in Back Sound, NC as the height at which the reef is subaerial 20-40% of the time. Compared to subtidal reefs, the intertidal position creates conditions more favorable for oyster growth, leading to increased production of biogenic sediments and nitrogen burial (Hogan & Reidenbach, 2021). The intertidal position may also enhance the capture of allogenic sediments, as higher relief may provide a greater surface area that intercepts waters that carry sediments.

Despite similar positions within the tidal frame, intertidal fringing oyster reefs exhibited lower relief values and buried nitrogen at significantly lower rates than intertidal sand flat reefs. Bost et al. (2021), found that salt marsh fringing reefs exhibit lower mean crest elevations and experience less aerial exposure time compared to patch reefs on sand flats. They discuss how the adjacent salt marsh can slow flow rates on the landward edge of fringing reefs and attenuate the flow of seston, producing less favorable conditions for biogenic sediment accumulation and vertical accretion. Moreover, fringing reefs' positions near the lower boundary of the OGZ (Bost et al., 2021), may explain the observed lower densities and possibly lower rates of biogenic sediment production.

Density generally scaled positively with N burial rates; however, we observed a maximum occurring at 3,000 individuals m^{-2}. The parabolic relationship observed between density and burial rates may reflect differences between habitat contexts. Intertidal fringing reefs accumulate relatively high amounts of N at relatively low densities. It may be that proximity to salt marsh, another depositional environment, simultaneously reduces flow rates and supplies additional sediments on the reef's landward edge, allowing material accumulation in the interstitial spaces of the fringing reef. In contrast, intertidal sand flat reefs exhibit high densities and N burial rates plateau. In this setting, erosional and depositional forces may be balanced differently such that oyster recruits, rather than sediments, fill the interstitial space (Ridge et al., 2015).

Proximity to vegetation also significantly affected sediment contents in the restored oyster reefs. Fringing reef sediments exhibited lower C:N ratios than other habitats contexts, meaning they bury proportionally more N than other habitat contexts. Differences in sediment sources and biogeochemical processes in the TAZ likely explain the observed differences in sediment contents. Westbrook et al. (2018) discuss elevated N concentrations in shallow water reefs compared to deep-water reefs in LA, citing inputs from productive marshes. It is possible that in Back Sound, NC, marshes augment the N supply in their adjacent fringing reefs, compared to habitat contexts more distal to vegetation. Differences in flow regime and organic

matter source/flux between intertidal fringing and sand flat reefs may indirectly affect sediment C:N ratios by affecting the phytoplankton availability in seston (Carmichael & Valiela, 2005). Wave baffling and slowed flow rates on the landward edge of fringing oyster reefs (Bost et al., 2021) may favor growth of benthic microalgae, which has been correlated with high biodeposit quality (lower C:N ratio; Hoellein et al., 2014). Another possible explanation for different C:N ratios in intertidal fringing vs sand flat reef habitat contexts is nitrogen removal through additional biogeochemical processes, such as denitrification. Smyth et al. (2015) reported oyster-enhanced denitrification in estuarine flat sediments, but not in salt marsh sediments. It's possible that nitrogen removal in the surrounding sand flat sediments (via coupled nitrification-denitrification) reduces the proportion of nitrogen in accumulated allogenic sediments and results in higher C:N ratios relative to fringing reefs.

Another important consideration is site-specific water chemistry, particularly ambient nitrate concentrations, as they may affect processes in the TAZ and ultimately the nitrogen pool available for burial (Hoellein et al., 2014). Smyth et al (2015) showed that oyster-mediated denitrification was significantly higher under elevated nitrate conditions compared to ambient concentrations. This is likely due to the increased contribution of direct denitrification, in which nitrate is extracted from the water column, contrasting coupled nitrification-denitrification in which nitrate is supplied by the sediments (Seitzinger et al., 2006). Chronically nitrate-enriched environments, such as waters downstream of a wastewater treatment facility (Smiley et al., 2025), may provide conditions that favor nitrogen removal from the water column via direct denitrification, allowing a larger pool of sediment nitrogen to be buried. This interpretation supports spatially targeting restoration sites can maximize nitrogen removal via multiple mechanisms.

4.3 Opportunities to inform restoration practices

Restoration costs are highly variable, ranging between $3,820 and $2,180,000 with an average cost of $299,999 USD per hectare (Bersoza Hernández et al., 2018) depending on project site, scale, and substrate (Bersoza Hernández et al., 2018; Blomberg et al., 2018; Gilby et al., 2017; Hall & DeAngelis, 2024). This variability paired with an incomplete understanding of ecosystem value makes estimating returns on investment difficult. Our measurements of nitrogen burial contribute to a more comprehensive understanding of ecosystem function and value. We valued nitrogen burial by oyster reefs at approximately $1,700 USD per hectare per year. While this figure may only be 0.05% of average restoration costs, annual returns are cumulative—over 20 years, a restored reef may recover 10% of restoration costs—and contribute to the larger supply of provisionary, regulatory, and cultural ecosystem services. Grabowski et al. (2012) estimated the annual value of nitrogen removal services—including denitrification and phytoplankton consumption— from oysters to be between $4,050 and $6,716 per hectare per year without accounting for removal via burial. Integrating our measurements of nitrogen burial could increase this figure by 25-42%.

The results of this study revealed that oyster reef restoration can be an effective strategy to reduce water column nitrogen through burial and should include consideration of habitat context. In Back Sound, NC, intertidal oyster reefs buried nitrogen at the highest rates and should be prioritized in restoration efforts and considered in concert with denitrification potential when the goal is nitrogen removal. Oyster reef restoration/construction plans should consider relief, ensuring that new reefs exist within the OGZ to promote sediment accumulation and resilience to sea level rise. Subtidal reefs produced the lowest average nitrogen burial rate of the three habitat contexts and likely exists below a critical exposure boundary under which growth rates will not keep up with sea level rise (Ridge et al., 2015). However, of the three habitat contexts evaluated in this study, subtidal reefs are the most abundant in Carteret County and provide the greatest overall economic value.

In addition to relief, restoration projects should also consider oyster densities to maximize water quality benefits. Intertidal oyster reefs (sand flat and fringing) exhibited a parabolic relationship between density and N burial, with a maximum occurring at 3,000 individuals m^{-2} and Smyth et al. (2015) identified a threshold of approximately 2,400 individuals m^{-2} for maximum denitrification. Dame et al. (2002) reported higher growth and recruitment rates in East Coast creeks where oysters had been removed. Oyster reefs continue to grow and remove nitrogen through burial and denitrification past these thresholds, but removal rates are slightly reduced. It's possible that these reefs exhibit a carrying capacity above which competition for space and nutrients limits growth and consequently production of nitrogen-rich biodeposits. Creating conditions that would enhance growth rates could also increase nitrogen removal through bioassimilation and extraction (Bricker et al., 2020; Carmichael et al., 2012).

Living shoreline construction is a common restoration practice and adaptive management strategy that typically include an intertidal fringing oyster reef adjacent to a marsh (Bilkovic et al., 2016). We found that these types of reefs bury nitrogen rapidly at relatively low densities and bury proportionally more nitrogen than reefs in other habitat contexts. Onorevole et al. (2018) demonstrated that living shoreline habitats in Bogue Sound, NC effectively remove nitrogen via denitrification at relatively young ages. Moreover, fringing reefs and their adjacent salt marshes each bolster ecosystem functions in the other. Examples include increased denitrification in marsh sediments under nitrate enriched conditions (Smyth et al., 2012) and enhanced carbon burial in marshes when oysters are present (Ridge et al., 2017). Furthermore, their position at the land water margin physically armors the shoreline to protect against erosion during storms (Meyer et al., 1997; Scyphers et al., 2011; Smith et al., 2018).

These data highlight how managing restoration schemes across multiple ecosystem services requires consideration. For instance, Fodrie et al. (2017) showed that in terms of carbon removal, intertidal (flat) reefs are the 'worst' option–subtidal could be preferrable as service provider. In terms of juvenile fishes, Grabowski et al. (2005) and Ziegler et al. (2017) showed that intertidal flat reefs augment densities, but for larger sub-adult and adult fishes, saltmarsh fringing appear to be more valuable, possibly due to marsh-reef connectivity (Fodrie et al. 2015).

Optimal reef design is, thus, ultimately dependent on the objectives of those restoring them, with these findings contributing to a more comprehensive understanding of oyster reef-driven processes to achieve those objectives.

Acknowledgements

This research was supported by the UNC Institute for the Environment, the National Science Foundation (OCE-1233327, OCE-1155628, OCE-1635950), the United States Coastal Research Program (W912HZ-22-COO-11), and North Carolina Sea Grant (12-HCE-20).

References

Aoki, L. R., McGlathery, K. J., & Oreska, M. P. J. (2019). Seagrass restoration reestablishes the coastal nitrogen filter through enhanced burial. *Limnology and Oceanography*, *65*(1), 1-12. https://doi.org/10.1002/lno.11241

Bahr, L. N., & Lanier, W. P. (1981). The ecology of intertidal oyster reefs of the south Atlantic coast: a community profile. *U.S. Fish and Wildlife Service, Office of Biological Services, Washington, D.C.*(FWS/OBS-81/15), 105.

Barbier, E. B., Hacker, S. D., Kennedy, C., Koch, E. W., Stier, A. C., & Silliman, B. R. (2011). The value of estuarine and coastal ecosystem services. *Ecological Monographs*, *81*(2), 169-193.

Bayraktarov, E., Saunders, M. I., Abdullah, S., Mills, M., Beher, J., Possingham, H. P., Mumby, P. J., & Lovelock, C. E. (2016). The cost and feasibility of marine coastal restoration. *Ecological Applications*, *26*(4), 1055-1074. https://doi.org/10.1890/15-1077

Beck, M. W., Brumbaugh, R. D., Airoldi, L., Carranza, A., Coen, L. D., Crawford, C., Defeo, O., Edgar, G. J., Hancock, B., Kay, M. C., Lenihan, H. S., Luckenbach, M. W., Toropova, C. L., Zhang, G., & Guo, X. (2011). Oyster reefs at risk and recommendations for conservation, restoration, and management. *BioScience*, *61*(2), 107-116. https://doi.org/10.1525/bio.2011.61.2.5

BenDor, T. K., Spurlock, D., Woodruff, S. C., & Olander, L. (2017). A research agenda for ecosystem services in American environmental and land use planning. *Cities*, *60*, 260-271. https://doi.org/10.1016/j.cities.2016.09.006

Bersoza Hernández, A., Brumbaugh, R. D., Frederick, P., Grizzle, R., Luckenbach, M. W., Peterson, C. H., & Angelini, C. (2018). Restoring the eastern oyster: how much progress has been made in 53 years? *Frontiers in Ecology and the Environment*, *16*(8), 463-471. https://doi.org/10.1002/fee.1935

Beseres Pollack, J., Yoskowitz, D., Kim, H. C., & Montagna, P. A. (2013). Role and value of nitrogen regulation provided by oysters (Crassostrea virginica) in the Mission-Aransas Estuary, Texas, USA. *PLoS One*, *8*(6), e65314. https://doi.org/10.1371/journal.pone.0065314

Bilkovic, D. M., Mitchell, M., Mason, P., & Duhring, K. (2016). The role of living shorelines as estuarine habitat conservation strategies. *Coastal Management*, *44*(3), 161-174. https://doi.org/10.1080/08920753.2016.1160201

Blomberg, B. N., Beseres Pollack, J., Montagna, P. A., & Yoskowitz, D. (2018). Evaluating the U.S Estuary Restoration Act to inform resoration policy implementation: A case study focusing on oyster reef projects. *Marine Policy*, *91*, 161-166.

Bost, M. C., Rodriguez, A. B., Ridge, J. T., Miller, C. B., & Fegley, S. R. (2021). Natural intertidal oyster reef growth across two landscape settings and tidal ranges. *Estuaries and Coasts*, *44*(8), 2118-2131. https://doi.org/10.1007/s12237-021-00925-2

Boynton, W. R., & Kemp, W. M. (1985). Nutrient regeneration and oxygen consumption by sediments along an estuarine salinity gradient. *Marine Ecology Progress Series*, *23*(1), 45-55.

Bricker, S. B., Grizzle, R. E., Trowbridge, P., Rose, J. M., Ferreira, J. G., Wellman, K., Zhu, C., Galimany, E., Wikfors, G. H., Saurel, C., Miller, R. L., Wands, J., Rheault, R., Steinberg, J., Jacob, A. P., Davenport, E. D., Ayvazian, S., Chintala, M., & Tedesco, M. A. (2020). Bioextractive removal of nitrogen by oysters in Great Bay Piscataqua River Estuary, New Hampshire, USA. *Estuaries and Coasts*, *43*, 23-38. https://doi.org/10.1007/s12237-019-00661-8

Burdige, D. J., & Zheng, S. (2003). The biogeochemical cycling of dissolved organic nitrogen in estuarine sediments. *Limnology and Oceanography*, *43*(8), 1796-1813. https://doi.org/10.4319/lo.1998.43.8.1796

Byers, J. E., Grabowski, J. H., Piehler, M. F., Hughes, A. R., Weiskel, H. W., Malek, J. C., & Kimbro, D. L. (2015). Geographic variation in intertidal oyster reef properties and the influence of tidal prism. *Limnology and Oceanography*, *60*(3), 1051-1063. https://doi.org/10.1002/lno.10073

Carmichael, R. H., & Valiela, I. (2005). Coupling of near-bottom seston and surface sediment composition: Changes with nutrient enrichment and implications for estuarine food supply and biogeochemical processing. *Limnology and Oceanography*, *50*(1), 97-105. https://doi.org/10.4319/lo.2005.50.1.0097

Carmichael, R. H., Walton, W., Clark, H., & Ramcharan, C. (2012). Bivalve-enhanced nitrogen removal from coastal estuaries. *Canadian Journal of Fisheries and Aquatic Sciences*, *69*(7), 1131-1149. https://doi.org/10.1139/f2012-057

Casas, S. M., La Peyre, J., & La Peyre, M. K. (2015). Restoration of oyster reefs in an estuarine lake: population dynamics and shell accretion. *Marine Ecology Progress Series*, *524*, 171-184. https://doi.org/10.3354/meps11198

Chambers, L. G., Gaspar, S. A., Pilato, C. J., Steinmuller, H. E., McCarthy, K. J., Sacks, P. E., & Walters, L. J. (2017). How well do restored intertidal oyster reefs support key biogeochemical properties in a coastal lagoon? *Estuaries and Coasts*, *41*(3), 784-799. https://doi.org/10.1007/s12237-017-0311-5

Coen, L. D., Brumbaugh, R. D., Bushek, D., Grizzle, R., Luckenbach, M. W., Posey, M. H., Powers, S. P., & Tolley, S. G. (2007). Ecosystem services related to oyster restoration. *Marine Ecology Progress Series*, *341*, 303-307.

Craft, C. (2007). Freshwater input structures soil properties, vertical accretion and nutrient accumulation of Georgia and US tidal marshes. *Limnology and Oceanography*, *52*(3), 1220-1230.

DeLaune, R. D., Reddy, C. N., & Patrick, W. H. (1981). Accumulation of plant nutrients and heavy metals through sedimentation processes and accretion in a Louisiana salt marsh. *Estuaries*, *4*, 328-334.

Fodrie, F. J., Rodriguez, A. B., Baillie, C. J., Brodeur, M. C., Coleman, S. E., Gittman, R. K., Keller, D. A., Kenworthy, M. D., Poray, A. K., Ridge, J. T., Theuerkauf, E. J., Lindquist, N. L., & Arnott, S. (2014). Classic paradigms in a novel environment: inserting food web and productivity lessons from rocky shores and saltmarshes into biogenic reef restoration. *Journal of Applied Ecology*, *51*(5), 1314-1325. https://doi.org/10.1111/1365-2664.12276

Gilby, B. L., Olds, A. D., Peterson, C. H., Connolly, R. M., Voss, C. M., Bishop, M. J., Elliott, M., Grabowski, J. H., Ortodossi, N. L., & Schlacher, T. A. (2017). Maximizing the benefits of oyster reefs restoration for finfish and their fisheries. *Fish and Fisheries*, *19*(5), 931-947. https://doi.org/10.1111/faf.12301

Grabowski, J. H., Baillie, C. J., Baukus, A., Carlyle, R., Fodrie, F. J., Gittman, R. K., Hughes, A. R., Kimbro, D. L., Lee, J., Lenihan, H. S., Powers, S. P., & Sullivan, K. (2022). Fish and invertebrate use of

restored vs. natural oyster reefs in a shallow temperate latitude estuary. *Ecosphere*, *13*(5). https://doi.org/10.1002/ecs2.4035

Grabowski, J. H., Brumbaugh, R. D., Conrad, R. F., Keeler, A. G., Opaluch, J. J., Peterson, C. H., Piehler, M. F., Powers, S. P., & Smyth, A. R. (2012). Economic valuation of ecosystem services provided by oyster reefs. *BioScience*, *62*(10), 900-909. https://doi.org/10.1525/bio.2012.62.10.10

Grabowski, J. H., Hughes, A. R., Kimbro, D. L., & Dolan, M. A. (2005). How Habitat Setting Influences Restored Oyster Reef Communities. *Ecology*, *86*(7), 1926-1935. https://doi.org/10.1890/04-0690

Hall, E., & DeAngelis, B. M. (2024). The business of oyster restoration: using traditional market-based approaches to estimate the oyster restoration economy. *Restoration Ecology*, *32*(5). https://doi.org/10.1111/rec.14143

Higgins, C. B., Stephenson, K., & Brown, B. L. (2011). Nutrient bioassimilation capacity of aquacultured oysters: quantification of an ecosystem service. *Journal of Environmental Quality*, *40*(1), 271-277. https://doi.org/10.2134/jeq2010.0203

Hoellein, T. J., Zarnoch, C. B., & Grizzle, R. E. (2014). Eastern oyster (Crassostrea virginica) filtration, biodeposition, and sediment nitrogen cycling at two oyster reefs with contrasting water quality in Great Bay Estuary (New Hampshire, USA). *Biogeochemistry*, *122*(1), 113-129. https://doi.org/10.1007/s10533-014-0034-7

Hogan, S., & Reidenbach, M. A. (2021). Quantifying tradeoffs in ecosystem services under various oyster reefs restoration designs. *Estuaries and Coasts*, *45*(3), 677-690. https://doi.org/10.1007/s12237-021-01010-4

Housego, R. M., & Rosman, J. H. (2015). A model for understanding the effects of sediment dynamics on oyster reef development. *Estuaries and Coasts*, *39*(2), 495-509. https://doi.org/10.1007/s12237-015-9998-3

Hynes, S., Burger, R., Tudella, J., Norton, D., & Chen, W. (2022). Estimating the costs and benefits of protecting a coastal amenity from climate change-related hazards: Nature based solutions via oyster reef restoration versus grey infrastructure. *Ecological Economics*, *194*. https://doi.org/10.1016/j.ecolecon.2022.107349

Keller, D. A., Gittman, R. K., Brodeur, M. C., Kenworthy, M. D., Ridge, J. T., Yeager, L. A., Rodriguez, A. B., & Fodrie, F. J. (2019). Salt marsh shoreline geomorphology influences the success of restored oyster reefs and use by associated fauna. *Restoration Ecology*, *27*(6), 1429-1441. https://doi.org/10.1111/rec.12992

Kellogg, M. L., Smyth, A. R., Luckenbach, M. W., Carmichael, R. H., Brown, B. L., Cornwell, J. C., Piehler, M. F., Owens, M. S., Dalrymple, D. J., & Higgins, C. B. (2014). Use of oysters to mitigate eutrophication in coastal waters. *Estuarine, Coastal and Shelf Science*, *151*, 156-168. https://doi.org/10.1016/j.ecss.2014.09.025

Lenihan, H. S., Peterson, C. H., & Allen, J. M. (1996). Does flow speed also have a direct effect on growth of active suspension-feeders: An experimental test on oysters. *Limnology and Oceanography*, *41*(6), 1359-1366. https://doi.org/10.4319/lo.1996.41.6.1359

Loomis, M. J., & Craft, C. B. (2010). Carbon Sequestration and Nutrient (Nitrogen, Phosphorus) Accumulation in River-Dominated Tidal Marshes, Georgia, USA. *Soil Science Society of America Journal*, *74*(3), 1028-1036. https://doi.org/10.2136/sssaj2009.0171

Mehvar, S., Filatova, T., Dastgheib, A., De Ruyter van Steveninck, E., & Ranasinghe, R. (2018). Quantifying economic value of coastal ecosystem services: a review. *Journal of Marine Science and Engineering*, *6*(1). https://doi.org/10.3390/jmse6010005

Meyer, D. L., Townsend, E. C., & Thayer, G. W. (1997). Stabilization and erosion control value of oyster cultch for intertidal marsh. *Restoration Ecology*, *5*(1), 93-99. https://doi.org/10.1046/j.1526-100X.1997.09710.x

Newell, R. I. E., Fisher, T. R., Holyoke, R. R., & Cornwell, J. C. (2005, 4-9 October). Influence of eastern oysters on nitrogen and phosphorous regeneration in Chesapeake Bay, USA. In: *The Comparative Roles of Suspension-Feeders in Ecosystems*. Proceedings of the NATO Advanced Research Workshop on The Comparative Roles of Suspension-Feeders in Ecosystems Nida, Lithuania.

O'Beirn, F. X., Luckenbach, M. W., Nestlerode, J. A., & Coates, G. M. (2000). Toward design criteria in constructed oyster reefs: oyster recruitment as a function of substrate type and tidal height. *Journal of Shellfish Research*, *19*(1), 387-395.

Onorevole, K. M., Thompson, S. P., & Piehler, M. F. (2018). Living shorelines enhance nitrogen removal capacity over time. *Ecological Engineering*, *120*, 238-248. https://doi.org/10.1016/j.ecoleng.2018.05.017

Palinkas, C. M., & Cornwell, J. C. (2011). A Preliminary Sediment Budget for the Corsica River (MD): Improved Estimates of Nitrogen Burial and Implications for Restoration. *Estuaries and Coasts*, *35*(2), 546-558. https://doi.org/10.1007/s12237-011-9450-2

Perog, B. D., Bowers-Doerning, C., Lopez Ramirez, C. Y., Marks, A. N., Torres Jr, R. F., Wolfe, M. L., & Zacherl, D. C. (2023). Shell cover, rugosity, and tidal elevation impact native and non-indigenous oyster recruitment: Implications for reef ball design. *Ecological Engineering*, *192*. https://doi.org/10.1016/j.ecoleng.2023.106969

Piehler, M. F., & Smyth, A. R. (2011). Habitat-specific distinctions in estuarine denitrification affect both ecosystem function and services. *Ecosphere*, *2*(1), 1-16. https://doi.org/10.1890/es10-00082.1

Ridge, J. T., Rodriguez, A. B., & Fodrie, F. J. (2017). Salt marsh and fringing oyster reef transgression in a shallow temperate estuary: implications for restoration, conservation and blue carbon. *Estuaries and Coasts*, *40*(4), 1013-1027. https://doi.org/10.1007/s12237-016-0196-8

Ridge, J. T., Rodriguez, A. B., Joel Fodrie, F., Lindquist, N. L., Brodeur, M. C., Coleman, S. E., Grabowski, J. H., & Theuerkauf, E. J. (2015). Maximizing oyster-reef growth supports green infrastructure with accelerating sea-level rise. *Sci Rep*, *5*, 14785. https://doi.org/10.1038/srep14785

Rodriguez, A. B., Fodrie, F. J., Ridge, J. T., Lindquist, N. L., Theuerkauf, E. J., Coleman, S. E., Grabowski, J. H., Brodeur, M. C., Gittman, R. K., Keller, D. A., & Kenworthy, M. D. (2014). Oyster reefs can outpace sea-level rise. *Nature Climate Change*, *4*(6), 493-497. https://doi.org/10.1038/nclimate2216

Rose, J. M., Bricker, S. B., Tedesco, M. A., & Wikfors, G. H. (2014). A role for shellfish aquaculture in coastal nitrogen management. *Environmental Science and Technology*, *48*(5), 2519-2525. https://doi.org/10.1021/es4041336

Rosenzweig, B. R., Groffman, P. M., Zarnoch, C. B., Branco, B. F., Hartig, E. K., Fitzpatrick, J., Forgione, H. M., & Parris, A. (2018). Nitrogen regulation by natural systems in “unnatural” landscapes: denitrification in ultra-urban coastal ecosystems. *Ecosystem Health and Sustainability*, *4*(9), 205-224. https://doi.org/10.1080/20964129.2018.1527188

Schulte, D. M., Burke, R. P., & Lipcius, R. N. (2009). Unprecedented restoration of a native oyster metapopulation. *Science*, *325*(3944), 1124-1128.

Scyphers, S. B., Powers, S. P., Heck, K. L., Jr., & Byron, D. (2011). Oyster reefs as natural breakwaters mitigate shoreline loss and facilitate fisheries. *PLoS One*, *6*(8), e22396. https://doi.org/10.1371/journal.pone.0022396

Seitzinger, S., Harrison, J. A., Böhlke, J. K., Bouwman, A. F., Lowrance, R., Peterson, B., Tobias, C., & Drecht, G. V. (2006). Denitrification across landscapes and waterscapes: A synthesis. *Ecological Applications*, *16*(6), 2064-2090.

Seitzinger, S. P. (1987). Nitrogen biogeochemistry in an unpolluted estuary: the importance of benthic denitrification. *Marine Ecology Progress Series*, *41*(2), 177-186.

Smiley, A. M. H., Thompson, S. P., & Piehler, M. F. (2025). Chronic enrichment affects nitrogen removal in tidal freshwater river and estuarine creek sediments. *Journal of Environmental Quality*. https://doi.org/10.1002/jeq2.20674

Smith, C. J., DeLaune, R. D., & Patrick Jr., W. H. (1985). Fate of riverine nitrate entering an estuary: denitrification and nitrogen burial. *Estuaries*, *8*(1), 15-21.

Smith, C. S., Puckett, B., Gittman, R. K., & Peterson, C. H. (2018). Living shorelines enhanced the resilience of saltmarshes to Hurricane Matthew (2016). *Ecological Applications*, *28*(4), 871-877. https://doi.org/10.1002/eap.1722

Smyth, A. R., Piehler, M. F., Grabowski, J. H., & Frid, C. (2015). Habitat context influences nitrogen removal by restored oyster reefs. *Journal of Applied Ecology*, *52*(3), 716-725. https://doi.org/10.1111/1365-2664.12435

Smyth, A. R., Thompson, S. P., Siporin, K. N., Gardner, W. S., McCarthy, M. J., & Piehler, M. F. (2012). Assessing Nitrogen Dynamics Throughout the Estuarine Landscape. *Estuaries and Coasts*, *36*(1), 44-55. https://doi.org/10.1007/s12237-012-9554-3

Velinsky, D. J., Paudel, B., Quirk, T., Piehler, M., & Smyth, A. (2017). Salt marsh denitrification provides a significant nitrogen sink in Barnegat Bay, New Jersey. *Journal of Coastal Research*, *78*, 70-78. https://doi.org/10.2112/si78-007.1

Westbrook, P., Heffner, L., & La Peyre, M. K. (2018). Measuring carbon and nitrogen bioassimilation, burial, and denitrification contributions of oyster reefs in Gulf coast estuaries. *Marine Biology*, *166*(1). https://doi.org/10.1007/s00227-018-3449-1

Ziegler, S. L., Grabowski, J. H., Baillie, C. J., & Fodrie, F. J. (2017). Effects of landscape setting on oyster reef structure and function largely persist more than a decade post-restoration. *Restoration Ecology*, *26*(5), 933-942. https://doi.org/10.1111/rec.12651

zu Ermgassen, P. S. E., DeAngelis, B., Gair, J. R., Ermgassen, S. z., Baker, R., Daniels, A., MacDonald, T. C., Meckley, K., Powers, S., Ribera, M., Rozas, L. P., & Grabowski, J. H. (2021). Estimating and applying fish and invertebrate density and production enhancement from seagrass, salt marsh edge, and oyster reef nursery habitats in the Gulf of Mexico. *Estuaries and Coasts*, *44*(6), 1588-1603. https://doi.org/10.1007/s12237-021-00935-0

Fig1

Click here to access/download;Figure;Fig 1.eps

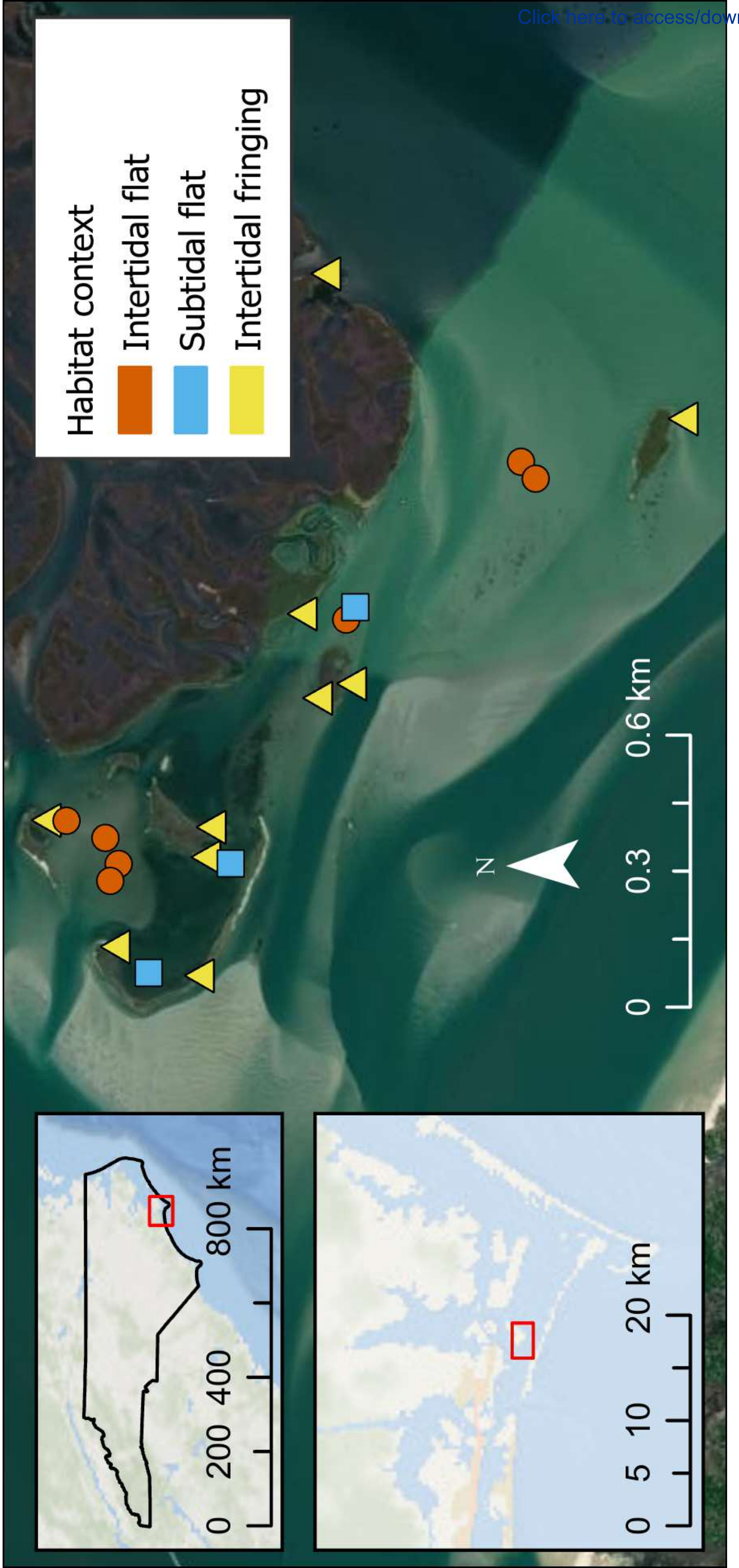

Fig2

Click here to access/download;Figure;Fig 2.eps

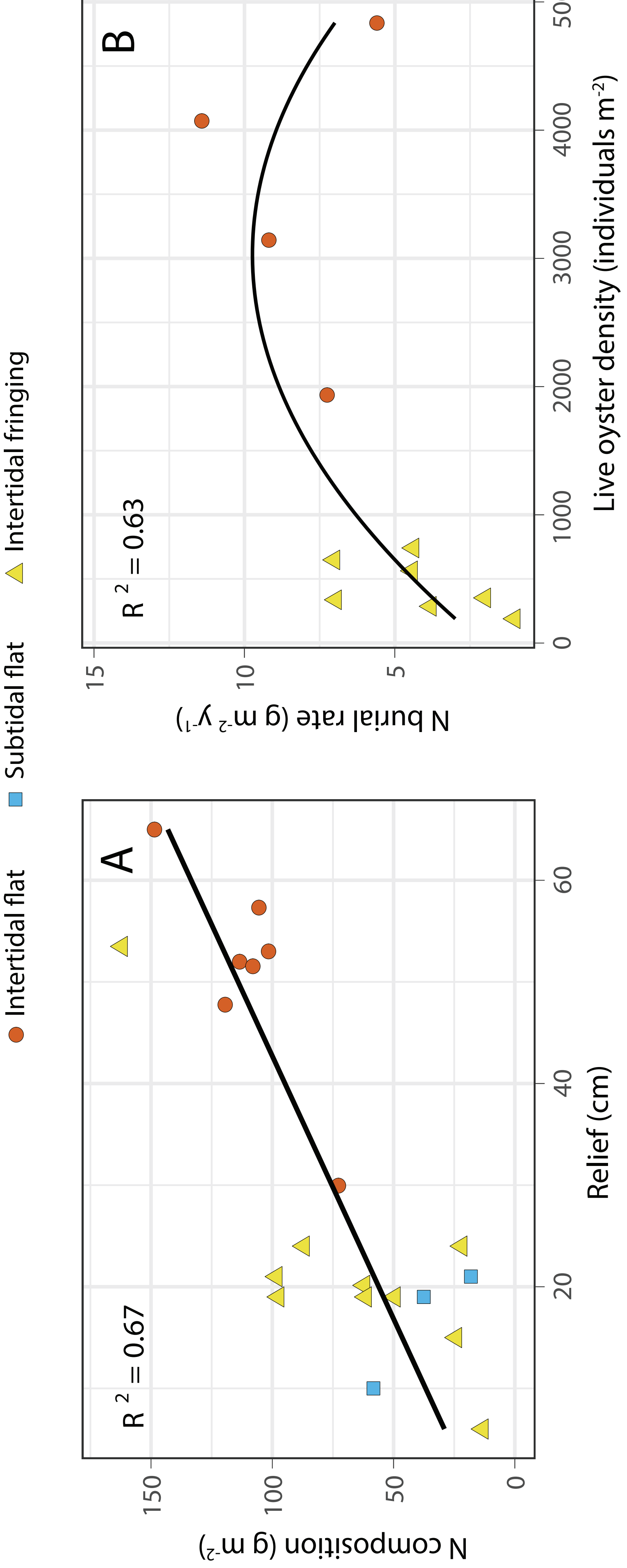

Fig3

Click here to access/download;Figure;Fig 3.eps

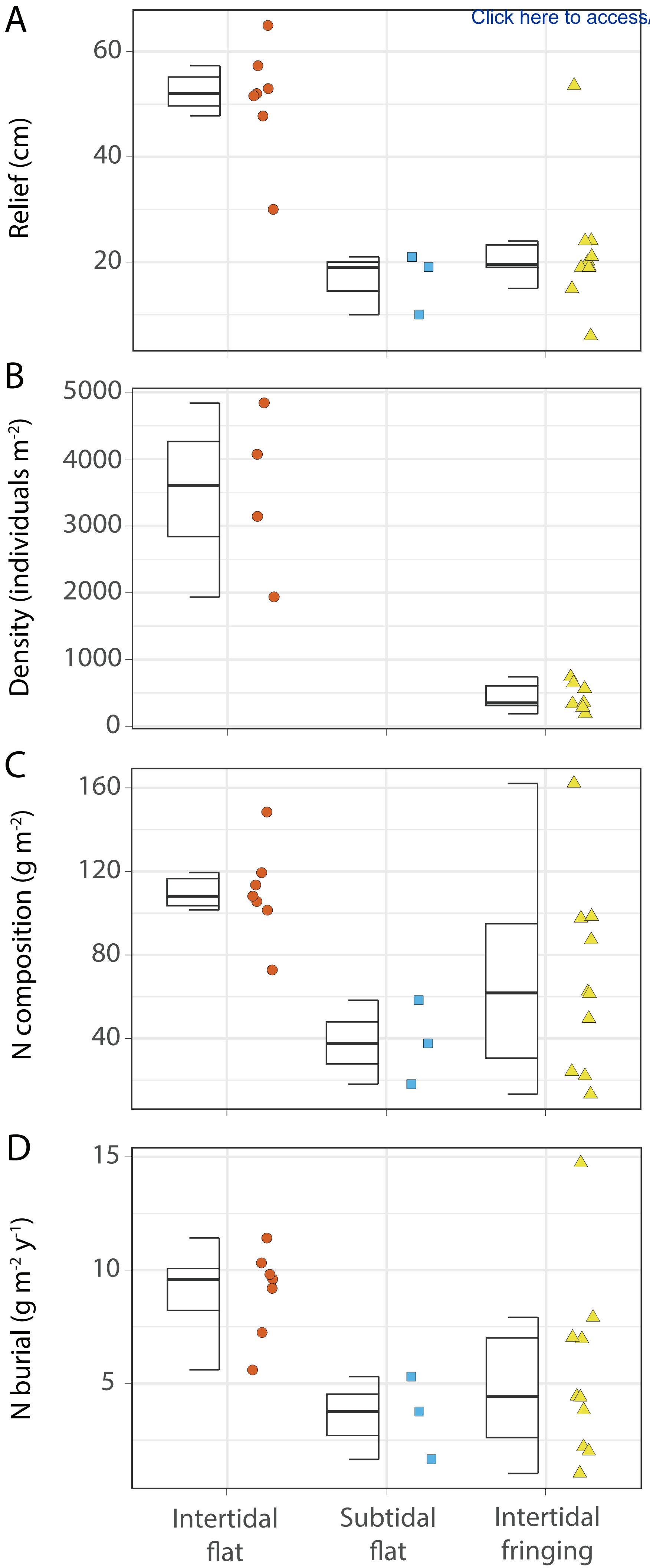

Fig4

Click here to access/download;Figure;Fig 4.eps

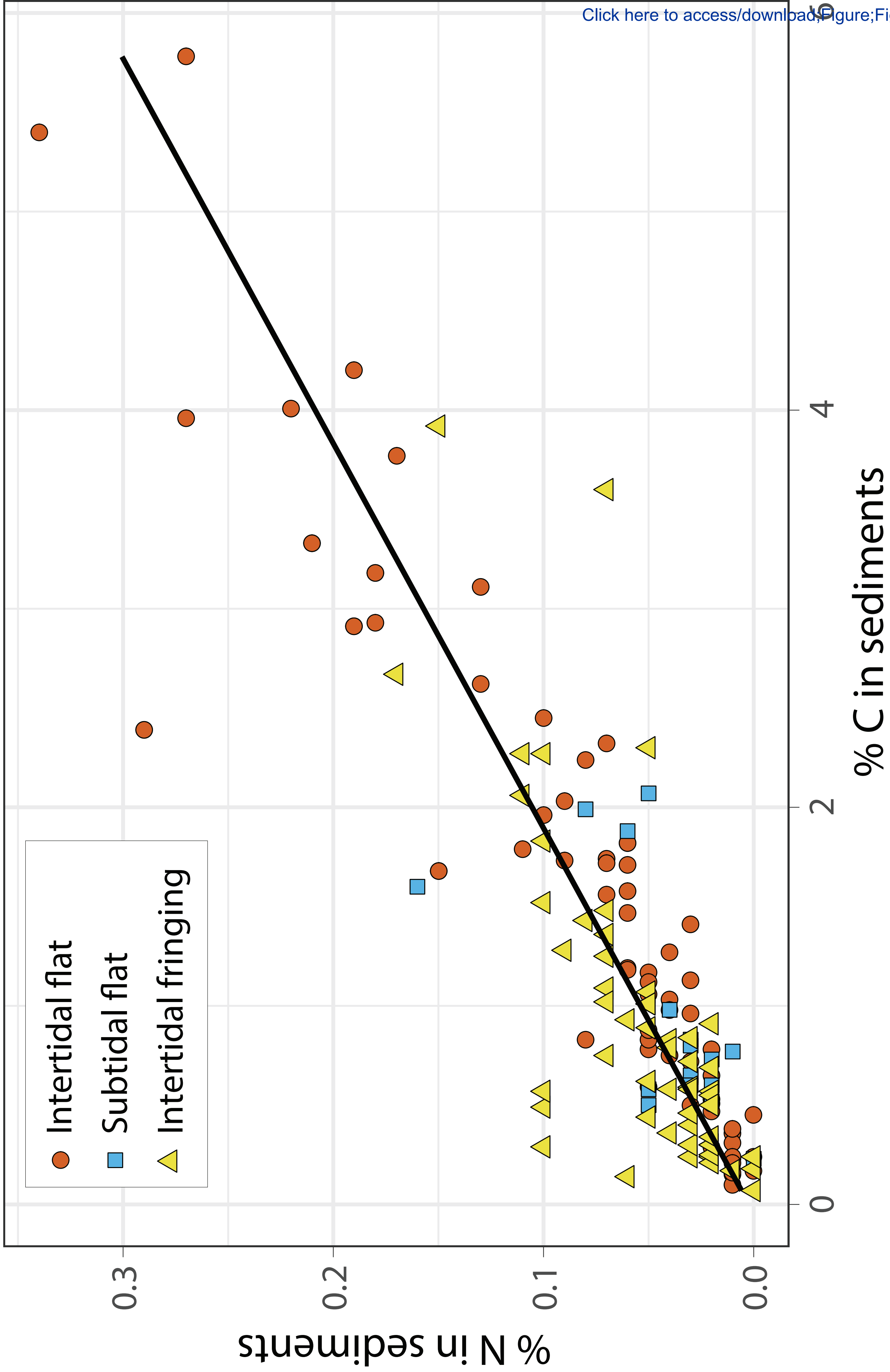

Fig5

Click here to access/download;Figure;Fig 5.eps

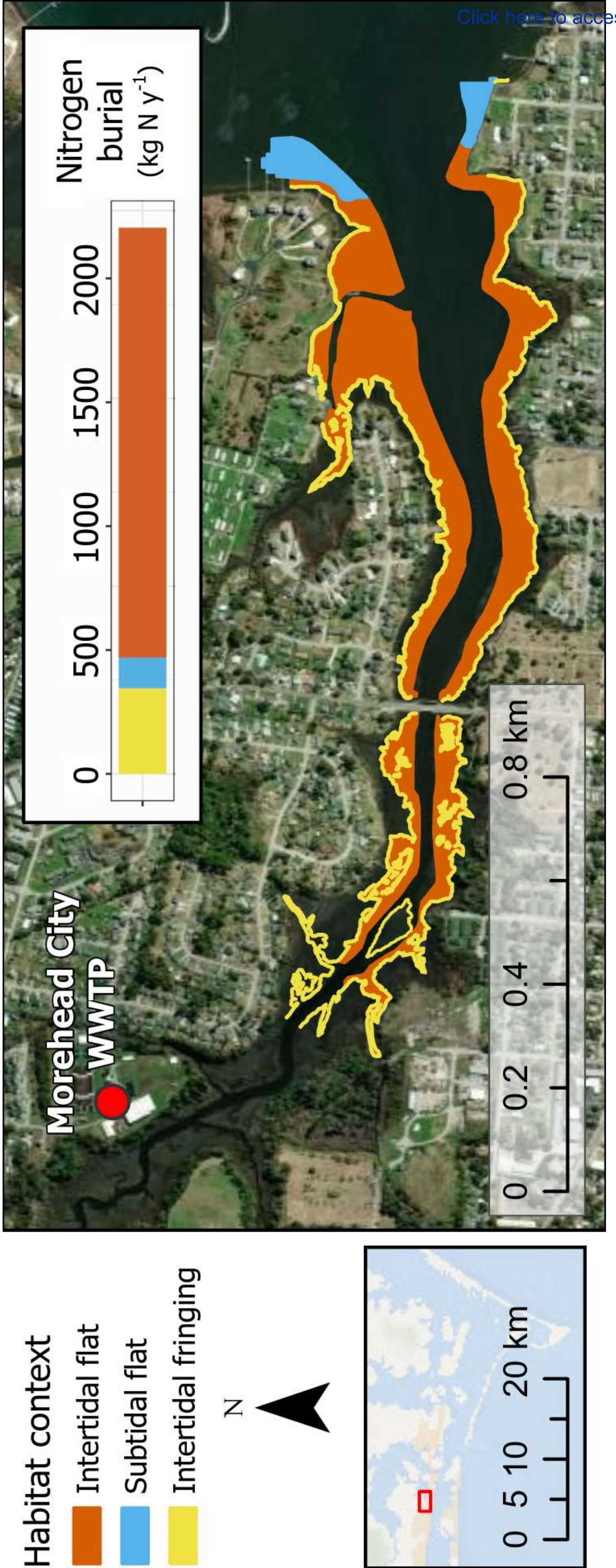

Supplement: Attachment — Submitted filename: PLOS ONE Reviewed.pdf [file pone.0344310.s001.pdf]
