# Supplementary material for: Habitat context affects sediment nitrogen burial by restored Eastern Oyster reefs
Source: PLoS One. 2026 Mar 25;21(3):e0344310. doi: 10.1371/journal.pone.0344310 (PMC13016323; doi:10.1371/journal.pone.0344310)
Supplement: Attachment — Submitted filename: Reviewer Responses.docx [file pone.0344310.s003.docx]

Reviewer #1: The manuscript presents a rigorous and timely study quantifying nitrogen burial rates across different habitat contexts in restored oyster reefs. It fills a key knowledge gap by directly measuring an overlooked process in nutrient cycling and ecosystem functioning. The design is robust, analyses are appropriate, and results are well supported. The discussion effectively integrates regional perspectives but could be more concise, particularly when comparing burial with denitrification. The manuscript is clearly written and scientifically sound, requiring only minor editorial refinement. Overall, it is a strong, policy-relevant contribution linking biogeochemical research, restoration practices, and ecosystem service valuation.

Reviewer #2: The study is well-structured and highlights meaningful differences across habitat contexts. However, some interpretations seem stronger than the supporting data allow, and a few assumptions—particularly around extrapolation and habitat equivalency require clearer justification or acknowledgment as limitations. Improving clarity in the methods, moderating broad claims, and more explicitly discussing uncertainty would strengthen the overall findings and their applicability to restoration planning. The author should also use more recent references.

*We would like to thank reviewers and editors for taking the time to read this manuscript and provide thoughtful feedback. Addressing these comments has improved the quality and clarity of this work. We have slightly restructured the Introduction and Discussion sections to improve the logical flow of themes and ideas. To do this, we combined the previously separate regional- and local-scale environmental context paragraphs/sections into one cohesive segment that relates environmental variability to structural differences in oyster reefs, then translated those structural differences to differences in functioning (nitrogen burial). We also provide additional methodological details as well as further explanation regarding spatial extrapolation, being more explicit about assumptions made and limitations of the methods. We include line-by-line response to specific comments below.*

Responses to specific editor comments:

1. Please ensure that your manuscript meets PLOS ONE's style requirements, including those for file naming. The PLOS ONE style templates can be found at

<https://journals.plos.org/plosone/s/file?id=wjVg/PLOSOne_formatting_sample_main_body.pdf> and

<https://journals.plos.org/plosone/s/file?id=ba62/PLOSOne_formatting_sample_title_authors_affiliations.pdf>

*Response: Thank you, we have adjusted the formatting of the manuscript to adhere to PLoS ONE’s style requirements.*

2. Thank you for stating the following financial disclosure:
This research was supported by the UNC Institute for the Environment, the National Science Foundation (OCE-1233327, OCE-1155628, OCE-1635950), the United States Coastal Research Program (W912HZ-22-COO-11), and North Carolina Sea Grant (12-HCE-20).
Please state what role the funders took in the study. If the funders had no role, please state: "The funders had no role in study design, data collection and analysis, decision to publish, or preparation of the manuscript."
If this statement is not correct you must amend it as needed.
Please include this amended Role of Funder statement in your cover letter; we will change the online submission form on your behalf.

*Response: Thank you. Please add the statement "The funders had no role in study design, data collection and analysis, decision to publish, or preparation of the manuscript."*

3. Thank you for stating the following in the Acknowledgments Section of your manuscript:
This research was supported by the UNC Institute for the Environment, the National Science Foundation (OCE-1233327, OCE-1155628, OCE-1635950), the United States Coastal Research Program (W912HZ-22-COO-11), and North Carolina Sea Grant (12-HCE-20).
We note that you have provided funding information that is not currently declared in your Funding Statement. However, funding information should not appear in the Acknowledgments section or other areas of your manuscript. We will only publish funding information present in the Funding Statement section of the online submission form.
Please remove any funding-related text from the manuscript and let us know how you would like to update your Funding Statement. Currently, your Funding Statement reads as follows:
This research was supported by the UNC Institute for the Environment, the National Science Foundation (OCE-1233327, OCE-1155628, OCE-1635950), the United States Coastal Research Program (W912HZ-22-COO-11), and North Carolina Sea Grant (12-HCE-20).

Please include your amended statements within your cover letter; we will change the online submission form on your behalf.

*Response: Thank you. We have removed all funding-related language from the manuscript. We have added the following text to the Acknowledgements section (lines 404-405): “We would like to think the field and laboratory teams that were instrumental in restored reef construction as well as sample collection and processing.”*

4. When completing the data availability statement of the submission form, you indicated that you will make your data available on acceptance. We strongly recommend all authors decide on a data sharing plan before acceptance, as the process can be lengthy and hold up publication timelines. Please note that, though access restrictions are acceptable now, your entire data will need to be made freely accessible if your manuscript is accepted for publication. This policy applies to all data except where public deposition would breach compliance with the protocol approved by your research ethics board. If you are unable to adhere to our open data policy, please kindly revise your statement to explain your reasoning and we will seek the editor's input on an exemption. Please be assured that, once you have provided your new statement, the assessment of your exemption will not hold up the peer review process.

*Response: Thank you. We have made all raw data included in our analyses available in a public repository hosted by the University of North Carolina at Chapel Hill at the following URL: https://cdr.lib.unc.edu/concern/data_sets/gq67k604s*

5. Please amend the manuscript submission data (via Edit Submission) to include author F. Joel Fodrie

*Response: Thank you. The list of authors includes F. Joel Fodrie as second author.*

6. Please amend your authorship list in your manuscript file to include author Joel Fodrie

*Response: Thank you. The list of authors includes F. Joel Fodrie as second author.*

7. Please include a caption for figure 5.

*Response: Thank you. the Caption for figure 5 is included and reads: “Fig 5. Map of oyster reefs in Calico Creek, NC, USA, as delineated by the NC Department of Marine Fisheries. Average nitrogen burial rates for each habitat context were multiplied by the area of respective reefs to estimate annual removal and value of the ecosystem service.”*

8. We note that Figures 1 and 5 in your submission contain map/satellite images which may be copyrighted. All PLOS content is published under the Creative Commons Attribution License (CC BY 4.0), which means that the manuscript, images, and Supporting Information files will be freely available online, and any third party is permitted to access, download, copy, distribute, and use these materials in any way, even commercially, with proper attribution. For these reasons, we cannot publish previously copyrighted maps or satellite images created using proprietary data, such as Google software (Google Maps, Street View, and Earth). For more information, see our copyright guidelines: <http://journals.plos.org/plosone/s/licenses-and-copyright>.

We require you to either (a) present written permission from the copyright holder to publish these figures specifically under the CC BY 4.0 license, or (b) remove the figures from your submission:

*Response: Thank you. Both figures were created using stock basemaps in ArcGIS Pro, and have been updated to include service layer credits.*

9. If the reviewer comments include a recommendation to cite specific previously published works, please review and evaluate these publications to determine whether they are relevant and should be cited. There is no requirement to cite these works unless the editor has indicated otherwise.

*Response: Thank you. The Reviewers did not recommend specific references. We did however, include additional, more recent citations upon Reviewer recommendation.*

10. Please review your reference list to ensure that it is complete and correct. If you have cited papers that have been retracted, please include the rationale for doing so in the manuscript text, or remove these references and replace them with relevant current references. Any changes to the reference list should be mentioned in the rebuttal letter that accompanies your revised manuscript. If you need to cite a retracted article, indicate the article’s retracted status in the References list and also include a citation and full reference for the retraction notice.

*Response: Thank you. We have checked that all published work mentioned in the text are included in the references and properly formatted.*

Responses to specific reviewer comments:

Line 18: Briefly mention the methods used

*Response: Thank you for this suggestion, this change has been made. Lines 17-18 in the abstract now read: “Elemental analysis of deep-core sediments revealed that nitrogen burial rates ranged between…”*

Line 21: How much? (in reference to C:N ratios)

*Response: Thank you, this addition has been made. Line 21 in the abstract now reads: “Intertidal fringing reefs exhibited the lowest mean C:N ratio, 18.5 ± 1.3—burying proportionally more nitrogen than reefs in other habitat contexts”*

Line 31: More recent citations need to be used, where possible. Generally, the introduction is too long and unfocused. Streamline by grouping content into 3–4 focused themes, directly connected to the study. Consolidate repetitive concepts for a better flow, such as ecosystem services and reef restoration. Same concepts are repeated multiple times at different points in the intro. Logical flow leading to the study objectives is needed.

*Response: Thank you for this feedback. We have streamlined the introduction and improved connecting sentences to improve flow, highlight fewer main ideas, and convey the following logical progression:*

*1) Importance of oysters and potential for resilient nature-based solutions [due to their ability to capture and retain sediments]*

*2) Overview of nitrogen processing in oyster reef sediments, highlighting poor understanding of burial*

*3) Environmental variables that influence reef structure and function, necessary information for effective restoration*

*4) Quantifying value of ecosystem services can inform cost-effective restoration*

*5) Overview of objectives/study.*

*The following specific changes have been made to the introduction:*

*Lines 33-34: Removed “contributions to ecosystem function through the provision of habitat and nursery areas, sediment stabilization, and nutrient cycling. These functions provide”*

*Lines 35-37: Rearranged this sentence to read “following historical losses—up to 85% globally (Beck et al., 2011)—increased oyster reef restoration in recent decades has attempted to reestablish populations and valuable ecosystem services (Bersoza Hernandez et al., 2018).”*

*Lines 37-39: Shortened and rearranged this sentence to read “Importantly, oysters’ ability to capture, produce, and retain sediments enable them to keep pace with sea level rise…”*

*Line 39: Added a broader context statement “…distinguishing reefs as intrinsically resilient nature-based solutions for coastal communities on the frontlines of change.”*

*Lines 41-43: Shortened this sentence to read “…deposition of feces and pseudofeces link oyster reef accretion to water quality.”*

*Line 47-48: Changed “could be an important sink” to “has been suggested as an important sink”.*

*Lines 50-58: Combined what were previously two separate paragraphs on regional and local environmental variables to broadly encompass environmental variables that affect reef properties and processes. We also removed the section about physicochemical characteristics of the water column to be more concise and added a closing sentence to emphasize the importance of understanding environmental controls on nitrogen processing. The single paragraph now reads:*

*“Environmental conditions, such as subaerial exposure, flow velocity, and adjacent habitats (Byers et al., 2015; Keller et al., 2019) can influence oyster reef properties and functioning. Reef relief and vertical position in the water column can affect oyster recruitment (O'Beirn et al., 2000; Perog et al., 2023; Schulte et al., 2009) and potentially reef assimilation of nitrogen and/or production of nitrogen-rich biodeposits. Exposure to high flow velocities has a strong influence on reef size and complexity (Bahr & Lanier, 1981), and surface area for sediment capture. Proximity to other estuarine habitats, such as marshes, can influence sediment flux and composition (Ridge et al., 2017), biogeochemical processing in the TAZ, and ultimately burial. A comprehensive understanding of environmental variables affecting ecological function is essential for effective oyster reef restoration.”*

*Lines 65-67: Changed text to emphasize the need for this study. It now reads “Notably, these figures do not include nitrogen removal through burial, highlighting the need to better quantify this process.”*

*Line 72: Changed “3” to “three”*

Line 33: Reference to this statement.

*Response: Thank you, the sentence focused on ecosystem function has been removed, but the sentence focused on ecosystem services and the associated references remain.*

Line 54: This paragraph is dense and confusing. Break into shorter sentences and clearly explain why burial is distinct from other pathways and why it matters.

*Response: Thank you for this suggestion. We maintain that a brief overview of nitrogen cycling in oyster reef sediments is important context, as we draw comparisons to additional nitrogen processes in the discussion. We distinguish nitrogen burial as the terminal process that is poorly understood compared to processes in the dynamic taphonomically active zone that are well characterized in comparison. To highlight this knowledge gap, this paragraph now ends with “Burial is oyster reef sediments has been suggested as an important sink for nitrogen (Beseres Pollack et al., 2013; Newell et al., 2005), but direct measurements of burial rates in these habitats are lacking.” (lines 47-49)*

Lines 90-91: Add a bridging statement explaining why these habitat contexts are expected to differ in nitrogen burial.

*Response: Thank you for this suggestion. We added “Positions within the tidal frame and relative to vegetation likely influence the capture, processing, and burial of nitrogen.”*

Line 128: Provide clear explanation why. (in refence to lack of subtidal density data)

*Response: Thank you for this comment. We provided this explanation on lines 111-113: “Density measurements were not collected on subtidal reefs as quadrat sampling could not be done in a manner consistent with sampling at intertidal reefs; including them would compromise comparability across habitat contexts.”*

Line 136: Exact number of cores per reef? How cores were handled (sealed? frozen? refrigerated?)

*Response: Thank you for this comment, we agree adding this information would improve clarity and reproducibility. On line 115, we specified that one sediment core was collected from the X-Y center of each reef. We also added to lines 118-122 “Cores were sealed and transported to the Institute of Marine Sciences in Morehead City, NC, where they were stored upright in a 2 degree C walk-in freezer. Within a week from collection, cores were sectioned into 5-cm increments, which were then ground, fumed…”*

Line 141: Specify equation

*Response: Thank you. We have specified the equation as:*

$$Burial rate= \frac{1}{Reef age}\sum_{i=1}^{n} {Bulk weight}_{i}*\frac{{\%N}_{i}}{100}$$

Line 165: Extrapolating burial rates to all county reefs assumes restored and natural reefs are equivalent, but the mapping data do not distinguish them. This may bias large-scale estimates and should be acknowledged or justified.

*Response: Thank you. We agree that it’s important to acknowledge limitations associated with scaling site-level data to larger areas. In this paragraph (line 170), we commented on the lack of distinction between natural and restored reefs, citing Chambers et al. (2017), which concludes that within one year restored reefs reach sediment nutrient concentrations comparable to natural reefs. Therefore, we assume our data from restored reefs are representative of rates in natural reefs. We did clarify at the end of this paragraph (lines 174-175) that a limitation of this dataset “is that it does not distinguish between reefs that are protected or closed to harvest from those that are actively harvested.”*

Line 228: The results present numerous pairwise comparisons but occasionally lack post-hoc p-values or effect sizes, making the interpretation of “significant” vs. “not significant” differences harder to follow.

*Response: Thank you for pointing this out. We have added R^2^ and p-values throughout the results section to improve consistency and clarity.*

Line 249: Relying on mean burial rates aggregated across habitats, may mask large differences between habitat types. It would be clearer to report habitat-specific economic values more explicitly before providing combined totals.

*Response: Thank you for this comment. We agree that it is important to distinguish habitat-context-specific contributions to the overall supply and value of nitrogen burial ecosystem services. We do not want to over-generalize our results; however, we thought a region-wide average would be a useful metric for coastal habitat managers and could be compared to additional regions. We moved the aggregated burial rate to the end of the paragraph to emphasize the heterogeneity of values before summarizing across treatments.*

Line 263: The discussion is too long and occasionally reads like an extended results section. Focus more on interpretation rather than repeating numerical values. Use latest references.

*Response: Thank you for this feedback. We have edited the discussion to be more concise and to improve flow. Most notably, we have combined the previously separate “Regional scale environmental context…” and “Local scale environmental context…” subsections into one subsection more broadly titled “Environmental context affects nitrogen burial”. We have restructured this section to begin with environmental variables that affect reef structure, then progress to how, at a habitat-context scale, those environmental conditions and structural differences translate to nitrogen burial rates.*

Lines: 268-280: This is just listing of studies. Provide interpretation, relevant to the results of this study.

*Response: Thank you for this feedback. We think it is important to place our burial measurements in the context of other structured habitats. In the first paragraph of the discussion, we compare our measurements to those measured in vegetated habitats in intertidal and subtidal positions—explaining that oyster reefs bury more nitrogen because their rough three-dimensional structure promotes sediment capture. We also thought it was important to contextualize burial rates with parts of nitrogen cycle that have been well characterized and used to assign value to oyster reefs in the same system (Back Sound, NC). We made the following specific changes to strengthen the connection between previous studies to the interpretation of our results:*

*Lines 262-263 (end of first discussion paragraph): added “Thus, to maximize nitrogen burial in estuaries, restoration of oyster reefs should be prioritized over other structured habitats.”*

*Lines 264-265 (beginning of second discussion paragraph): added “Nitrogen removal in oyster reefs is an important sink, comparable with other nitrogen removal mechanisms”*

*Lines 265-266: changed text to read “Mean nitrogen burial measured in this study fall within the range reported by Smyth et al. (2012) for average annual denitrification rates in Bogue Sound—5-7* *g N m^-2^ y^-1^.”*

*Lines 267-269: changed text to read “Compared with bioassimilation of nitrogen into oyster tissues and subsequent harvest, nitrogen removal via burial can be up to five times greater; Beseres Pollack et al. (2013) estimated that oyster reefs in the Mission-Aransas Estuary can remove roughly 1.2 g N m^-2^ y^-1^ through harvest.”*

*Line 270: added “Accounting for nitrogen removal burial will significantly increase previous value estimate of nitrogen removal services provided by oyster reefs.”*

Lines 297-298: Support with reference.

*Response: Thank you. This sentence has been slightly changed to read “Nitrogen burial was strongly correlated with reef relief and generally scaled positively with density; thus, active removal of individuals may hamper the capacity to bury nitrogen.” (290-292) to directly connect harvest pressure to our observations.*

Line 401: Clarify whether your data align with the 2,400–3,000 ind. m⁻² range.

*Response: Thank you. The density values originally included in the results sections were mistakenly the raw data from the 0.25 m^2^ quadrats. The data are reported in units m^2^ and those values should have been multiplied 4. This oversight has been corrected and aligns with the 2,400–3,000 ind. m⁻² range.*

Line 422: The final paragraph effectively highlights trade-offs, but it might benefit from a concluding sentence summarizing how nitrogen burial fits within multi-service restoration planning.

*Response: Thank you. We agree that that this section and the manuscript would benefit from a stronger closing sentence. We have slightly adjusted the text and added additional in lines 398-402 text to read: “Optimal reef design is, thus, ultimately dependent on the restoration objectives, with these findings advancing an understanding of oyster-driven processes to achieve those objectives. By identifying environmental drivers of nitrogen burial, this work can inform effective coastal habitat and nutrient management to maximize water quality benefits.”*
